# A rapid IL-17 response to *Cryptosporidium parvum* in the bovine intestine

**DOI:** 10.1016/j.vetimm.2017.07.009

**Published:** 2017-09

**Authors:** Emma Drinkall, Matthew J. Wass, Tracey J. Coffey, Robin J. Flynn

**Affiliations:** a School of Veterinary Medicine & Science, University of Nottingham, Sutton Bonington, LE12 5RD, United Kingdom; b Department of Infection Biology, Institute of Infection and Global Health, University of Liverpool, L3 5RF, United Kingdom

**Keywords:** *Cryptosporidium parvum*, Biopsy, IL-17A, Intestine, Villi, Bovine

## Abstract

*Cryptosporidium parvum* causes diarrhoea, due to villi damage, in livestock and humans globally. Immunity develops after repeated infections but initial infections can be severe, highlighting the importance of early infection dynamics. We have modelled early *C. parvum* infection in bovine jejunum biopsies. IL-17A accumulated over time peaking at 9 h post-infection, with no effect of infection on IL-1β; antibiotics positively influenced IL-17A as higher levels were found in cultures with antibiotics. Infection of primary fibroblasts resulted in lower plaque formation when fibroblasts were primed with IL-17A. Our results indicate a role for IL-17A in reducing *C. parvum*-dependent host cell damage.

## 1 Introduction

Diarrhoea is one of the most common neonatal clinical signs of disease encountered in calves, and in the United Kingdom it is thought to be a factor in approximately 50% of calf death with over one third of calves being affected at some point (Morrill et al., 2012). There are several significant infectious causes of neonatal diarrhoea in calves including the protozoan parasite Cryptosporidium (NADIS, 2017). Infection with *Cryptosporidium parvum* was shown to be the likely sole cause in over 20% of cases, and also contributed to the pathology of mixed infections of alternative pathogens (NADIS, 2017). *C. parvum* infection begins upon ingestion of oocysts from which sporozoites emerge, the parasite remains extracellular despite merging with the host cell membrane to form a parasitophorous vacuole. Sporozoites transform to asexual trophozoites before repeated rounds of replication resulting in the formation of microgamonts and macrogamonts that fuse to give rise to infectious oocysts. *C. parvum* infection causes villous atrophy, through a loss of enterocytes in the villi, which causes them to recede in order to maintain a continuous epithelial barrier. The precise sequence of mechanisms of enterocyte loss is unknown, but is thought to involve apoptosis (Pollok et al., 2001). As enterocytes are lost there is a progressive shortening of the gut villi, eventually giving rise to the malabsorptive diarrhoea that is indicative of infection (Foster and Smith, 2009).

An early robust response is clearly required to prevent excessive cell death and thus facilitate a reduction in severe or fatal diarrhoea. Identification of *C. parvum* via *myd88* is essential as knock-out mice harboured greater parasite burdens (Rogers et al., 2006). Additionally, in SCID mice there was strong IFNα/β expression 24 h after infection suggestive of a local innate intestinal response independent of the adaptive immune response (Barakat et al., 2009a). Destruction of the epithelium is important as it is a critical source of cyotkines such as IL-18 which has been shown to play a role in the activation of IFN-γ production from NK cells (Choudhry et al., 2012). Moreover natural killer (NK) cells were shown to aid in the resistance to infection when mice lacking *rag2* were infected (Barakat et al., 2009b), again indicting a role of the innate response. The cross-species importance of NK cells was further demonstrated in an ovine model of disease where within 6 days post-infection (pi) activation of NK cells was increased, despite no overall increase in cell numbers, with a concomitant increase in perforin expression – a known effector against intracellular parasites (Olsen et al., 2015).

IL-17, and its other family members, are a family of cytokines shown to play important roles in performing effector functions or regulating inflammatory states in a number of protozoan infections (Kelly et al., 2005, Weaver et al., 2007). We have previously shown a role for bovine IL-17 in the control of the related parasite *Neospora caninum* (Peckham et al., 2014). Two single studies of IL-17 during Cryptosporidium infection exist to date. Zhao et al. (2014) found an increase in IL-17 mRNA levels within 12 h pi in chickens, and a study of *C. parvum* in mice found that within 6 h pi IL-17 mRNA was upregulated in the intestine and within 24 h in the spleen (Zhao et al., 2016). These studies clearly suggest that IL-17 signalling is rapidly upregulated early in infection. Herein we sought to examine the dynamics of early *C. parvum* infection in a bovine gut model.

## 2 Materials and methods

### 2.1 Gut biopsy culture

Gut tissues were obtained from a commercial abattoir in County Derbyshire, UK, following ethical approval of the study by the School of Veterinary Medicine and Science, 18 month old Belgian Blue bulls (N = 3) destined for the human food chain were selected. A 6 mm biopsy punch was used to obtain a full width biopsy of the jejunum immediately post-mortem. These were washed in PBS (Sigma-Aldrich) and transported in sterile PBS to the laboratory within 3 h of collection. *Cryptosporidium parvum* oocysts (Creative Science Company, Moredun) were excysted by incubating them in 0.025% trypsin (Sigma-Aldrich) in acidified water (pH 2.4) at 37 °C for 20 min. The solution was then centrifuged at 1800*g* for 10 min and the supernatant removed. Oocysts were prepared in both abx-free and abx [penicillin 100 U/mL and streptomycin 100 ng/mL] containing media (RPMI 1640 plus 10% heat-inactivated FCS − all Sigma-Aldrich) at a concentration of 2 × 10^5^ sporozoites/mL. At sampling points 1 h, 3 h, 6 h, 9 h, 12 h and 18 h during the culture period, tissue biopsies were fixed in 10% neutral buffered formalin (Sigma Aldrich). Tissue blocks were sectioned and trimmed at 0.5 μm, mounted on polysine-coated slides and stained with haematoxylin eosin. Sections were examined at 5× magnification on a Leica DM5000B microscope. One field of view of the biopsy for each time point was examined and 3 different villi lengths were recorded.

### 2.2 Cytokine ELISA and LDH measurement

Media from biopsy cultures were collected and stored at −20 °C. IL-1β (ThermoScientific ESS0027) and IL-17A (Kingfisher Biotech Ltd DIY0673B-003) protein levels were determined using commercial assays; protocols were conducted as per manufacturers’ guidelines. The lower limits of detection were 31 pg/mL and 95 pg/mL, respectively.

Lactate dehydrogenase (LDH) release was measured using a commercial kit from Promega. 50 μL of cell-free supernatant was added to a 96 well plate to which 50 μL of CytoTox reagent was added. Plates were incubated for 30 min at RT. 50 μL of stop solution was then added and optical density was recorded at 490 nm.

### 2.3 Fibroblast infection

Fibroblasts were prepared as previously described (Peckham et al., 2014) bovine skin biopsies were collected at post-mortem washed in D-PBS with 25 μg/mL amphotericin B, penicillin 100U/mL and streptomycin 100 ng/mL. Thereafter biopsies were trimmed to 5 mm × 5 mm size and placed in petri dishes with 10 mL of complete media. After 4 days the biopsy was removed and cells were washed, collected and re-seeded into 175 cm^2^ flasks until confluent. 2.16 × 10^4^ cells/mL were seeded into 24 well plates and cultured for 24 h at 37 °C. Plates were centrifuged and the supernatant was removed, fresh media alone or with varying concentrations of murine IL-17 (Peprotech; #210-17) was added and plates incubated for a further 24 h at 37 °C. Cells were then infected with *C. parvum* oocysts (see above for excystation conditions) at a concentration of 2.16 × 10^4^ sporozoites/mL for a final 24 h at 37 °C. The cells were stained with 3% Giemsa solution and any excess was removed by washing with distilled water immediately afterwards, before plaques, areas of cell death/damage, were manually counted on a light microscope (Leica DM5000 B microscope).

### 2.4 Statistical analysis

Raw data was collected in Excel workbooks (Microsoft) and analysis of data was conducted in Graphpad Version 6 (Prism). P values of <0.05 were taken as significant, and details of individual tests are provided in the figure legend.

## 3 Results and discussion

### 3.1 Infection of bovine gut biopsies causes rapid villi destruction

**Fig. 1:**
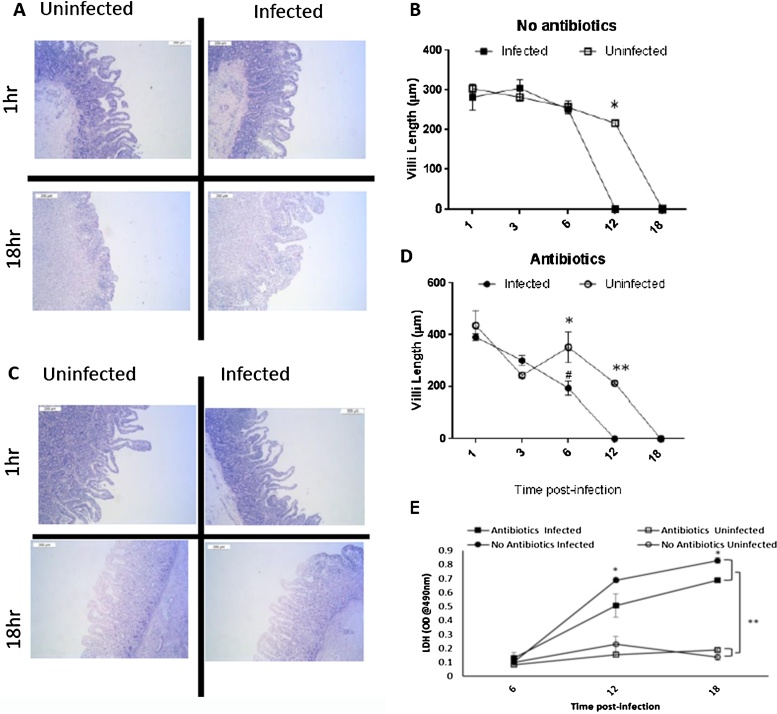
Dynamics of gut breakdown following *C. parvum* infection of bovine gut biopsies. A and C representative histology images from a single animal infected or not at 1hr or 18hr post-infection cultured in the absence (A) or presence of abx (C) scale bar represents 200 μm. (B) and (D) Changes in the length of villi were measured for three images for timepoint per animal for three individual animal, the graph displays mean ± SEM over time of three animals; (B) data quantified from no abx and (D) data quantified from cultures with abx. 2-way Anova was used to determine differences in villi length overtime in the presence of absence or Abx and *C. parvum*. Differences within abx treatments (Antibiotics or No antibiotics) are shown with *, where * P < 0.05 and **P < 0.01. Across the abx or no abx treatments a significant difference was found between C. parvum infected cultures at 6 h denoted by # P < 0.05. (E) LDH was quantified in the biopsy media at 6, 12, and 18 h and reported as OD@490 nm; 2-way Anova was used to determine the interaction between *C. parvum* infection or not and abx or not. For infected cultures there was a significant (*P<0.05) differences in the presence or absence or abx. Similarly, independent of abx there was an effect of *C. parvum* on LDH release.

Following infection, villous length was recorded for each time point. Autolysis was evident in all cultures from 12 h and complete autolysis and destruction of tissue with a loss of villi and even crypt structure is seen at 18 h (representative images in Fig. 1A and C). Villous length was found to decrease significantly over time (p < 0.001) (Fig. 1B and D). Shortening of the villi was greater and occurred faster in infected cultures compared to non-infected cultures indicated in Fig. 1B and D (*P < 0.05 and **P < 0.01). When comparing the absence or presence of abx; cultures without abx maintained normal villous lengths for longer during the culture when compared to culture with abx (# on Fig. 1D, p < 0.001). Confirming that infection with *C. parvum* will result in destructive changes in the intestine that shortens the villi length, and that these changes can over a short period be monitored *in vitro*. Significantly, in the presence of abx (penicillin/streptomycin) this damage is reduced in terms of the speed at which it takes place; suggesting that resident gram-positive and negative bacteria can have a buffering effect early during *C. parvum* infection. As a proxy measure of cell death we also assayed for LDH release in the media from the biopsy cultures at 6, 12, and 18 h. In both conditions with and without abx LDH accumulated over time and was greater in the presence of *C. parvum* (P < 0.01, Fig. 1E). Analysis of the mid-point of culture suggested that in the presence of abx *C. parvum* induced significantly lower levels of LDH compared to those *C. parum* cultures without abx (P < 0.05).

### 3.2 Cytokine production from infected biopsies

**Fig. 2:**
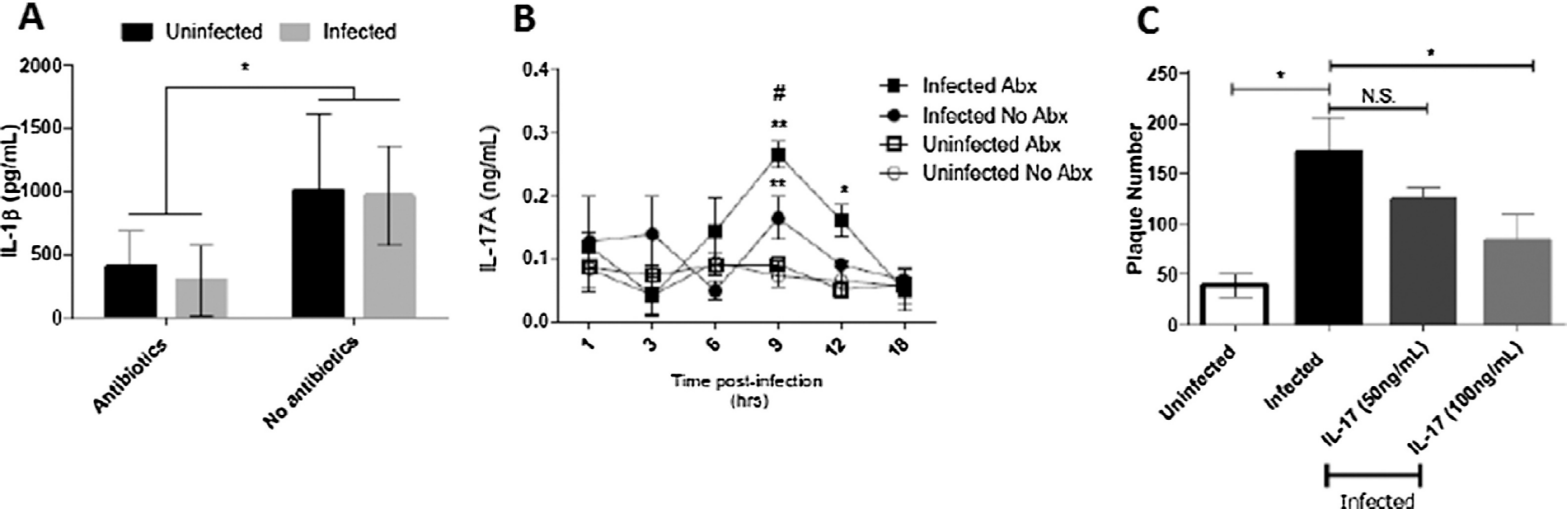
(A) IL-1β was measured in gut biopsy culture supernatant at 18 h post-infection by ELISA. There was no impact of infection on IL-1β but a significant increase following removal of antibiotics, tested by 1-way anova (*P < 0.05). (B) IL-17A kinetics, levels increased over time to a peak at 9 h. Levels were significantly elevated in all infected conditions compared to 1hr; 2-way anova **P < 0.01. Significant differences were also found between cultures with and without abx at 9 h #P < 0.05. Results are mean ± SEM of three animals. (C) Fibroblasts pre-treated with IL-17A had reduced plaque numbers following infection with *C. parvum* for 24 h, as judged by 1-way anova (*P < 0.05), results presented are mean ± SEM of three experiments.

IL-1β is rapidly processed and released upon recognition of pathogens by a pathway referred to as the inflammasome (Coccia et al., 2012); IL-1β was previously found in biopsies of C. parvum challenged volunteers (Robinson et al., 2001) and had a weak protective effect on HT-29 cells following infection (Pollok et al., 2001). To investigate potential causes of this observation effect we analyzed supernatants collected at 18 h post-infection for levels of IL-1β. We found abundant IL-1β in biopsies cultures but there was no effect of infection (Fig. 2A) despite a significant effect of abx as in their absence both infected and non-infected biopsies displayed a ∼2-fold, increase in IL-1β levels (Fig. 2A). This would suggest that in our biopsy model there was no role for IL-1β in mediating the villi shortening which is surprising given the diverse roles for IL1β in mediated infection induced tissue damage in the intestine (Coccia et al., 2012).

We next determined if IL-17A levels changed during infection, with or without abx, and found a significant increase (** p < 0.01) in levels at 9 h both with and without abx and 12 h only in infected biopsies with abx (* p < 0.05) (Fig. 2B). In the absence of abx IL-17A levels were significantly higher in cultures (# P < 0.05). The rapid induction of IL-17A protein following infection would suggest that a cell resident in the intestine is capable of doing so. We have previously shown that IL-17A secreting γδ T-cells could specifically target and reduce the intracellular parasite burden in infected fibroblasts (Peckham et al., 2014). We next sought to test a role for IL-17A in mediating direct protection against *C. parvum*. Obtaining autologous blood to match the donor of the intestine biopsy is logistically difficult, so co-culture experiments to demonstrate a role for γδ T-cells were not possible.

### 3.3 IL-17A protects fibroblasts against damage

We investigated a role for IL-17A in mediating direct protection against *C. parvum*. Healthy primary fibroblasts were cultured for 24 h in the presence of recombinant IL-17A (50 and 100 ng/mL). Following this cultures were infected and damage to the cell monolayer was assessed by counting plaques after a further 24 h. While there was no effect of 50 ng/mL of IL-17A we did detect a significant reduction in the number of plaques when cultures were pre-treated with 100 ng/mL of IL-17A (p < 0.05) (Fig. 2C). Thus our results would suggest that IL-17A can act directly on fibroblasts to reduce the number of plaques caused by infection − potentially limiting damage. A caveat to this finding is that the dose of IL-17A used here is much higher than the levels detected in our biopsy culture; this was necessary given our use of a murine recombinant protein in the absence of bovine recombinant at the time. Additionally, the levels of *ex vivo* or *in vitro* cytokine production has been shown to be a poor indicator of the *in vivo* levels of cytokine produced given the speed at which they are catabolized and used *in vivo* (Finkelman and Morris, 1999).

Given the rapid response in terms of IL-17A protein production it would suggest either resident leukocytes or the epithelium are responsible and that the direct effects of this could limit the tissue damage caused by *C. parvum*. Epithelial cells are known to be a source of IL-17A in diverse settings including infection and transplant rejection (Loverre et al., 2011). However, indirect effects of IL-17A may still play a role as it has been shown that IL-17A can induce antimicrobial peptides in *Helicobacter pylori* infection (Dixon et al., 2016). The presence of antibiotics increased the IL-17A response during infection. This would suggest that the interaction between resident bacteria and invading *C. parvum* is at least synergistic but likely more complex, as previous studies have shown in mice that the presence of segmented filamentous bacteria are essential for the development of IL-17 secreting cells within the intestine (Ivanov et al., 2009). Thus removal of resident bacteria or treatment symptoms of *C. parvum* infection with antibiotics may inadvertently alter the resident microflora and affect the outcome in terms of immune response. Given the recent publication of a study which suggests that IL-17A contributes to pathogenicity in malnourished murine hosts (Bartelt et al., 2016), our information may be useful in helping to guide treatment of bovine Cryptosporidiosis. The overall question as to whether IL-17A is protective in driving elimination of parasite-infected host cells remains to be determined, but the use neutralizing antibodies may allow for the dissection of this process. Given that IL-17A can also contribute to the generation of antibody responses, via T-follicular helper cells (Hirota et al., 2013), it remains to be seen if IL-17A will play a role in protective bovine antibody responses against *C. parvum* (Askari et al., 2016, Jenkins et al., 1999, Perryman et al., 1999).
